# Gray Matter Densities in Limbic Areas and APOE4 Independently Predict Cognitive Decline in Normal Brain Aging

**DOI:** 10.3389/fnagi.2019.00157

**Published:** 2019-06-28

**Authors:** François R. Herrmann, Cristelle Rodriguez, Sven Haller, Valentina Garibotto, Marie-Louise Montandon, Panteleimon Giannakopoulos

**Affiliations:** ^1^Department of Rehabilitation and Geriatrics, Division of Geriatrics, Geneva University Hospitals and University of Geneva, Geneva, Switzerland; ^2^Department of Psychiatry, University of Geneva, Geneva, Switzerland; ^3^Medical Direction, Geneva University Hospitals, Geneva, Switzerland; ^4^CIRD Centre d’Imagerie Rive Droite, Geneva, Switzerland; ^5^Department of Surgical Sciences, Radiology, Uppsala University, Uppsala, Sweden; ^6^Faculty of Medicine, University of Geneva, Geneva, Switzerland; ^7^Division of Nuclear Medicine and Molecular Imaging, Diagnostic Department, Geneva University Hospitals, Geneva, Switzerland

**Keywords:** longitudinal study, cognition, magnetic resonance imaging, gray matter density, white matter hyperintensity, arterial spin labeling, hippocampus, amygdala

## Abstract

Cross-sectional magnetic resonance imaging (MRI) studies reported significant associations between gray matter (GM) density changes in various limbic and neocortical areas and worst cognitive performances in elderly controls. Longitudinal studies in this field remain scarce and led to conflicting data. We report a clinico-radiological investigation of 380 cognitively preserved individuals who undergo neuropsychological assessment at baseline and after 18 months. All cases were assessed using a continuous cognitive score taking into account the global evolution of neuropsychological performances. The vast majority of Mini Mental State Examination (MMSE) 29 and 30 cases showed equal or worst performance at follow-up due to a ceiling effect. GM densities, white matter hyperintensities and arterial spin labeling (ASL) values were assessed in the hippocampus, amygdala, mesial temporal and parietal cortex at inclusion using 3 Tesla MRI Scans. Florbetapir positron emission tomography (PET) amyloid was available in a representative subsample of 64 cases. Regional amyloid uptake ratios (SUVr), mean cortical SUVr values (mcSUVr) and corresponding z-scores were calculated. Linear regression models were built to explore the association between the continuous cognitive score and imaging variables. The presence of an APOE-ε4 allele was negatively related to the continuous cognitive score. Among the areas studied, significant associations were found between GM densities in the hippocampus and amygdala but not mesial temporal and parietal areas and continuous cognitive score. Neither ASL values, Fazekas score nor mean and regional PET amyloid load was related to the cognitive score. In multivariate models, the presence of APOE-ε4 allele and GM densities in the hippocampus and amygdala were independently associated with worst cognitive evolution at follow-up. Our data support the idea that early GM damage in the hippocampus and amygdala occur long before the emergence of the very first signs of cognitive failure in brain aging.

## Introduction

Cognitive trajectories in old age are variable ranging from cognitive stability to fluctuations over time and, in a limited number of cases, progressive worsening of neuropsychological performances corresponding to the pre-mild cognitive impairment (MCI) state. In a large sample from the Alzheimer’s Disease Neuroimaging Initiative (ADNI) cohort followed-up annually for 5 years, 40% of cases showed stable and high memory and executive function performances (successful agers) whereas 20% displayed progressive decline (declining agers; Lin et al., 2017). Similar data were obtained in Mexican Americans (Downer et al., 2017) and Korean aged over 65 years (Min, 2018). Although within normal age-adjusted performances, declining agers are thought to exhibit the first signs of cognitive frailty and are of particular interest for future therapeutic interventions. Most of the previous investigations in this field focused on the concept of preclinical AD searching to establish the predictive power of lesion burden, functional and structural brain changes in community-based samples of elderly controls. In this context, a combination of AD biomarkers in cerebrospinal fluid (Aβ_42_, tau, and phospho-tau), non-invasive neuroimaging, and genetic risk factors have been investigated with promising but also conflicting data (for review, see Khan, 2018). When addressing the cognitive trajectories in old age, one should keep in mind the marked heterogeneity of elderly controls. Prior to 80 years, most of them did not correspond to the preclinical AD concept and their cognitive fate may be determined by a variety of other parameters such as significant vascular burden or limited cognitive reserve due to genetic or environmental factors (Li et al., 2017). But even within the theoretical framework of preclinical AD, there is substantial heterogeneity with respect to its neuropsychological definition (Epelbaum et al., 2017). Most of the longitudinal investigations in cognitively intact elderly individuals examined the evolution of neuropsychological parameters (working and episodic memory as well as executive abilities; Dubois et al., 2018; Rabin et al., 2018) or screening test (more frequently Mini Mental State Examination, MMSE) scores over time. However, cognitive performances may vary substantially in healthy elders with some of them declining and others remaining stable or even improving at follow-up. An accurate approach of cognitive evolution in this population needs to consider both improvement and decline of task performances in a wide range of cognitive tests over short time periods.

Among the different imaging techniques used to predict the cognitive evolution in elderly controls, amyloid positron emission tomography (PET) imaging and structural magnetic resonance imaging (MRI) changes are likely to address two distinct processes. Based on 1,209 cognitively intact individuals aged 50–95, Jack and collaborators showed that hippocampal volume loss may occur before abnormal amyloid PET occurrence. Unlike hippocampal volume decrease that starts at 30 years, and becomes significant after 60 years and is APOE4-independent, amyloid PET positivity occurred after 70 years and depended on the presence of APOEε4 alleles. These data indicated that Aβ accumulation arises in later life on a background of preexisting structural deficits that are associated with aging and not with amyloid pathology *per se* (Jack et al., 2015). Several observations pointed to the dissociation between neurodegeneration and amyloid pathology in normal aging and proposed two spatially distinct patterns of atrophy, a tau-related cortical thinning and Aβ-related hippocampal volume decrease, that may have a synergistic effect on subtle cognitive decline (Besson et al., 2015; Edmonds et al., 2015; Insel et al., 2015; Wang et al., 2015).

Given the recent progress in MRI analysis, several parameters are now available to explore the neuroanatomical substrates of the progressive transition from preserved cognition to the initial stages of cognitive deterioration. In normal aging, diffusion tensor imaging studies showed early fractional anisotropy decrease in the hippocampus and parahippocampal gyrus, supramarginal gyrus, frontotemporal lobes, mesial temporal lobes and anterior cigulate cortex (Hong et al., 2016; Lancaster et al., 2016). Data on gray matter (GM) are, however, more ambiguous. Regional GM decrements in right thalamus, left parahippocampal gyrus, inferior temporo-parietal lobules, anterior cingulum, and precentral gyrus have been documented (Lee et al., 2016; Fletcher et al., 2018; Squarzoni et al., 2018) but in certain cohorts GM densities were preserved or marginally affected in healthy controls (Hong et al., 2016; Takeuchi et al., 2017). The initial stages of cognitive deterioration may be related not only to structural but also functional changes that affect brain perfusion. In this line, we reported that reduced arterial spin labeling (ASL) in the posterior cingulate cortex at baseline is associated with the development of subtle neuropsychological deficits in healthy elderly controls (Xekardaki et al., 2015).

The present longitudinal study of a community-based cohort of highly educated elderly individuals explores the demographic, clinical and 3T MRI correlates of very subtle cognitive decline (prior to MCI) controlling for the presence of amyloid pathology. In order to obtain a global assessment of cognitive status without *a priori* hypotheses, we established a continuous cognitive score taking into account not only AD-related cognitive functions and considering both improvement and decline of neuropsychological performances at follow-up. Using univariate and multivariate linear regression models controlled for demographic variables (age, gender, education), MMSE scores at baseline, amyloid load, and Fazekas score of white matter lesion severity, we explored the association between subtle cognitive changes and patterns of GM volumes and ASL values in limbic and temporo-parietal areas (Bharath et al., 2017; Zanchi et al., 2017).

## Materials and Methods

### Population

The protocol was approved by the Ethics Committee of the Geneva University Hospitals of Geneva. All experimental procedures were carried out in accordance with the approved guidelines and with the principles of the Declaration of Helsinki. All participants were given written informed consent prior to inclusion. These community-based cases were recruited *via* advertisements in local newspapers and media. All participants had normal or corrected-to-normal visual acuity. Past hearing problems were identified as a part of the medical interview (including both subjects and their proxies). All cases with such problems were *a priori* excluded. Audition was tested by standard audiologic tests including self-report and speech in noise perception in all cases during clinical routine medical examination. Cases with self-report of hearing loss and altered speech in noise perception were addressed in specialized consultation and were not considered for further investigations. The education level was defined according to the Swiss scholar system, where level 1 = less than 9 years (primary school), level 2 = between 9 and 12 years (high school) and level 3 = more than 12 years (university). To control for the confounding effect of cardiovascular diseases, individuals with subtle cardiovascular symptoms and a history of stroke and transient ischemic episodes were also excluded from the present study. The inclusion period for control subjects was from October 2014 to March 2016.

The final sample included 380 elderly controls: 232 (61.1%) women and 148 (38.9%) men, aged 74.2 ± 4.1 (mean ± SD) ranging from 68.6 to 90.0 years, all assessed with structural and resting state fMRI at baseline (Zanchi et al., 2017).

### Neuropsychological Assessment

Participants were evaluated at inclusion with an extensive neuropsychological battery, including the MMSE (Folstein et al., 1975), the Hospital Anxiety and Depression Scale (HAD; Zigmond and Snaith, 1983), and the Lawton Instrumental Activities of Daily Living (IADL; Barberger-Gateau et al., 1992). Cognitive assessment included: (a) attention [Digit-Symbol-Coding (Wechsler, 1997a), Trail Making Test A (Reitan, 1958)]; (b) working memory [verbal: Digit Span Forward (Wechsler, 1997b), visuospatial: Visual Memory Span (Corsi; Milner, 1971)]; (c) episodic memory [verbal: RI-48 Cued Recall Test (Buschke et al., 1997), visual: Shapes Test (Baddley et al., 1994)]; (d) executive functions [Trail Making Test B (Reitan, 1958), Wisconsin Card Sorting Test (WCST; Heaton et al., 1993) and Phonemic Verbal Fluency Test (Cardebat et al., 1990)]; (e) language (Boston Naming Test; Kaplan et al., 1983); (f) visual gnosis (Ghent, 1956); and (g) praxis ideomotor (Schnider et al., 1997), reflexive (Poeck, 1985), and constructional (Consortium to Establish a Registry for Alzheimer’s Disease, CERAD), figures copy (Welsh et al., 1994).

In agreement with the criteria of Petersen et al. (2001), participants with a CDR score of 0.5 but no dementia and a score exceeding 1.5 standard deviations (SDs) below the age-appropriate mean in any of the previously mentioned tests were classified as MCI and were excluded. Participants who met DSM-IV diagnostic criteria of dementia on the basis of the neuropsychological and clinical assessments were also excluded. Participants with neither dementia nor MCI were classified as cognitively healthy older controls and underwent full neuropsychological assessment once at follow-up, on average 18 months later, with the same neuropsychological battery.

### APOE Assessment

Whole blood samples were collected at baseline for all subjects for APOE genotyping. Standard DNA extraction was performed using either 9 ml EDTA tubes (Sarstedt, Germany) or Oragene Saliva DNA Kit (DNA Genotek, Inc., Ottawa, ON, Canada) which were stored at −20°C. APOE genotyping was done on the LightCycler (Roche Diagnostics, Basel, Switzerland) as described previously (Nauck et al., 2000). Subjects were classified according to the presence of an APOEε4 allele (ε4/ε3, ε3/ε3, ε3/ε2 carrier).

### MRI Imaging

Imaging data were acquired on a 3T MRI Scanner (TRIO Siemens Medical Systems, Erlangen, Germany). A high-resolution T1-weighted anatomical scan (magnetization prepared rapid gradient echo (MPRAGE), 256 × 256 matrix, 176 slices 1 mm isotropic, TR = 2,300 ms, TE = 2.27 ms,) was collected as well as a pulsed ASL sequence [64 × 64 matrix, 24 slices, voxel size 3.44 × 3.44 × 5 mm^3^, TE 12 ms, TR 4,000 ms, inversion time (TI) 1,600 ms]. Additional sequences included axial fast spin-echo T2-weighted imaging (4,000/105, 30 sections, 4-mm section thickness), susceptibility weighted imaging (28/20, 208 × 256 × 128 matrix, 1 × 1 × 1 mm^3^ voxel size) were performed to exclude brain disease, such as ischemic stroke, subdural hematomas, or space-occupying lesions.

### Assessment of Gray Matter Volumes

3DT1 MRIs were preprocessed with the FSL software package^1^, according to the standard procedure. The essential processing steps included brain extraction with the FSL Brain Extraction Tool^2^, tissue-type segmentation with the FMRIB Automated Segmentation Tool^3^, nonlinear transformation into Montreal Neurological Institute reference space, and creation of a study-specific GM template to which the native GM images were then nonlinearly reregistered. The modulated segmented images were then smoothed with an isotropic Gaussian kernel with a width of 2 mm. Furthermore, we created a mask for the bilateral mesial temporal cortex, hippocampus, amygdala, caudate nuclei, and parietal lobes that was then applied to the GM image of the study-specific template, and we obtained GM density values in each area of interest.

### Arterial Spin Labeling

The reconstructed relCBF ASL perfusion images were spatially normalized using a linear spatial alignment from ASL raw data to the individual high-resolution 3DT1 image, followed by the application of the non-linear spatial registration determined in the pre-processing of the 3DT1 data. These spatial transformations were then applied to the relCBF maps calculated directly on the MRI scanner. This two-steps approach results in a non-linear spatial registration of the ASL relCBF map into the MNI space. We then calculated the relCBF values in each area of interest.

In addition to the GM and ASL analysis, white matter lesion severity was analyzed on T2-weighted images according to the established Fazekas scale (Fazekas et al., 1987).

### Amyloid PET Imaging With (18)F-Forbetapir

Florbetapir images were acquired 50–60 min after injection for 77 subjects. Seventy PET data were acquired on a Discovery PET/CT 710 scanner (GE Healthcare) and 7 PET imaging were performed using a Biograph™ mCT scanner (Siemens). All studies were quantitatively evaluated using the Amyvid BRASS commercial automated functional brain analysis software (Hermes Medical Solutions AB, Stockholm, Sweden). Regional amyloid uptake ratios (SUVr), mean cortical SUVr values (mcSUVr) and z-score (number of SDs from the healthy control SUVr in the BRASS template) were calculated relative to the cerebellum.

### Statistics

Demographic and neuropsychological data were compared between the two visits with paired *t*-test and Wilcoxon matched-pairs signed rank test. The significance level was set at *P* < 0.05 but was corrected to *P* < 0.0079 for multiple testing by using the Benjamini-Hochberg method (Benjamini and Hochberg, 1995).

The subtle cognitive decline continuous score was defined as follows. Most of the cognitive performances, discrete or continuous, cannot be linearly combined by adding the individual scores to a unique composite cognitive score. Thus all values were converted to z scores. Subsequently, we summed the number of cognitive tests at follow-up with performances at least 0.5 SD higher compared with the first evaluation, leading to the number of tests with improved performances (range, 0–14). Similarly, we summed the number of cognitive tests at follow-up with performances at least 0.5 SDs lower compared with the first evaluation, yielding the number of tests with decreased performances (range, 0–14). Finally, the number of tests with improved minus the number of tests with decreased performances results in a final continuous cognitive score.

Simple and multiple linear regression models were used to identify predictors of the continuous cognitive score (dependent variable) including GM volumes, ASL values and potential confounders such as age, gender, education levels, MMSE at baseline, Fazekas score, and APOE genotyping. Full models and models with only the significant variables were obtained by stepwise backward selection process. Each model was run for all four regions independently (hippocampus, middle temporal gyrus, parietal cortex, amygdala). To examine whether working memory or episodic memory changes were associated with our independent variables, we run the same regression models using the neuropsychological tests (z-score) used for these functions (Digit Span Forward, Visual Memory Span, RI-48 Cued Recall Test and Shapes Test) as the dependent variable. The same models were also run while adding normalized cortical amyloid volume assessed by florbetapir PET expressed either as a continuous variable or a binary score (below vs. above or equal to SUVr 1.2).

We also added to the above simple and multiple linear regression models an Apoe4*GM volume interaction term and subsequently built an ANCOVA model to perform a power analysis using the PASS version 13 software (PASS 13. NCSS, LLC. Kaysville, Utah, USA^4^, 2014).

All other statistics were performed with the STATA statistical software, Version 15.1 (StataCorp, College Station, TX, USA, 2017).

## Results

### Sample Description

**Figure 1:**
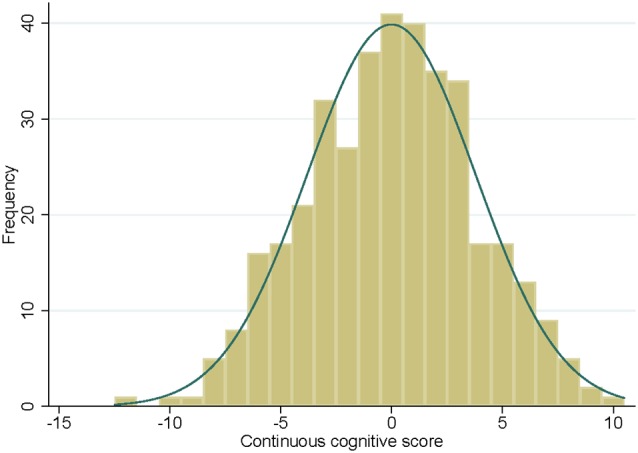
Histogram of the continuous cognitive score at follow-up with the corresponding normal density curve.

The distribution of the continuous cognitive score at follow-up is illustrated in Figure 1. Education levels were distributed as follows: 63 (16.6%) level 1 (primary school), 175 (46.0%) level 2 (high school) and 142 (37.4%) level 3 (university). Sixty-five (17.1%) participants had at least one allele APOEε4. Mean MMSE score was of 28.5 ± 1.3 (mean ± SD) ranging from 24.0 to 30.0.

### Cognition

**Figure 2:**
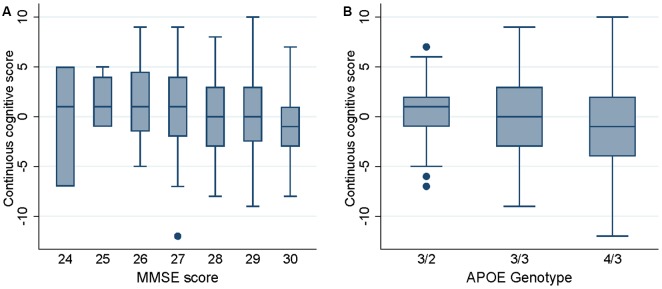
Box plots of the continuous cognitive score at follow-up as a function of Mini Mental State Examination (MMSE) score (panel **A**) and APOE genotype (panel **B**).

Univariate analysis showed that MMSE at baseline was negatively related to the continuous cognitive score [regression coefficient: −0.48 (−0.78, −0.17)] meaning that the highest is the MMSE score the lower is the cognitive score (implying that the most preserved cases are at higher risk to deteriorate; see Figure 2A). This analysis also showed that the presence of an APOEε4 allele was associated with significantly lower continuous cognitive scores [regression coefficient: −1.09 (−2.13, −0.04); see also Figure 2B]. Neither age nor gender or education was related to the cognitive outcome in this cohort.

### Cognition and GM Densities

**Figure 3:**
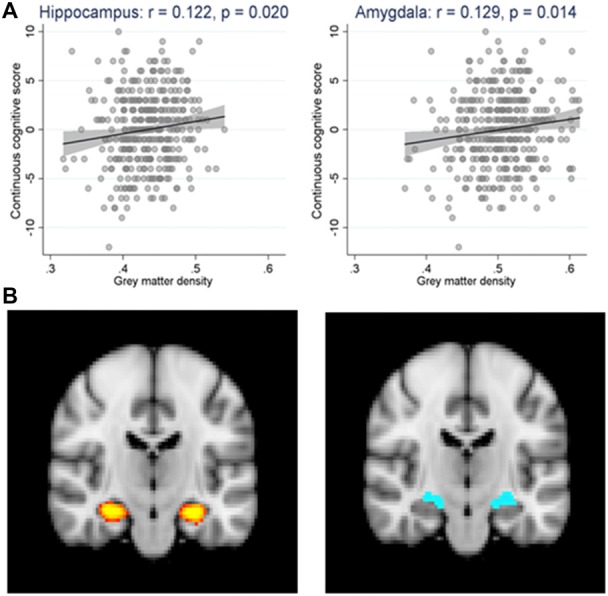
**(A)** Scatter plot and linear regression (along with its 95% confidence bands) of the continuous cognitive score (cgs) over the gray matter density (gmd) in the hippocampus (cgs = −5.44 + 12.55* gmd, *p* = 0.020) and the amygdala (cgs = −5.57 + 11.04* gmd, *p* = 0.014) regions. *N* = 362. *r* = Pearson’s correlation coefficient. **(B)** The regions of interest are highlighted in yellow for the hippocampus (left panel) and blue for the amygdala (right panel) in brain magnetic resonance imaging (MRI) scan.

Among the areas studied, significant positive associations were found between GM densities in the hippocampus and continuous cognitive score [regression coefficient: 12.54 (1.99, 23.10); see Figure 3 left panel]. This was also the case for the amygdala [regression coefficient: 11.04 (2.24, 19.84); see Figure 3 right panel] but not for mesial temporal and parietal gyrus. Importantly, ASL values in the areas studied were not related to the cognitive score. This was also the case for the Fazekas score in all of the areas studied.

In multivariate models taking into account MMSE scores, APOE genotyping, and the three MRI variables (GM densities, ASL values, Fazekas score), the presence of APOEε4 allele, higher MMSE score at baseline and GM densities in the hippocampus and amygdala were all associated with worst cognitive evolution at follow-up (Table 1). Although significant, the model including these parameters explained 7% of the cognitive variability. To explore a possible ceiling effect of MMSE score in our sample, we examined the association between MMSE scores at baseline and its evolution at follow up (increment or decrement; Table 2). The vast majority of MMSE 29 and 30 cases showed equal or worst performance at follow-up due to a ceiling effect. When working or episodic memory tests alone were used as dependent variable, no significant association was found with MRI variables (data not shown).

**Table 1:** Multiple linear regression models predicting the continuous cognitive score (dependent variable) with only the significant variables obtained by stepwise backward selection process and adjusted for the main confounders [Mini Mental State Examination (MMSE) at baseline, APOE genotyping, ASL, Gray matter index and FAZEKAS score].

	Reduced model				
Region	Coef	95% CI			P
HIPPOCAMPUS
APOE Genotype
3/2	0.219	−1.021	−	1.460	0.728
3/3	0.000				–
4/3	−1.051	−2.092	−	−0.011	0.048
MMSE	−0.499	−0.806	−	−0.191	0.002
ASL	0.002	−0.001	−	0.004	0.135
Gray matter index	12.701	2.090	−	23.311	0.019
FAZEKAS
Absent	0.000				–
Mild	0.419	−0.464	−	1.302	0.352
Moderate	0.804	−0.375	−	1.983	0.181
Severe	−0.327	−1.980	−	1.325	0.697
AMYGDALA
APOE Genotype
3/2	0.133	−1.112	−	1.377	0.834
3/3	0.000				–
4/3	−1.044	−2.079	−	−0.008	0.048
MMSE	−0.493	−0.800	−	−0.186	0.002
ASL	0.002	−0.0002	−	0.003	0.078
Gray matter index	10.575	1.699	−	19.452	0.020
FAZEKAS
Absent	0.000				–
Mild	0.432	−0.448	−	1.312	0.335
Moderate	0.748	−0.426	−	1.923	0.211
Severe	−0.292	−1.942	−	1.359	0.728

*A different model was run for each of the two regions*.

**Table 2:** Association between MMSE scores at baseline and its evolution at follow up (increment or decrement).

	Delta MMSE score (follow-up minus baseline)											
	Worsening						Improvement					
	−5	−4	−3	−2	−1	0	1	2	3	4	5	Total
Baseline MMSE												
24	0	0	0	0	0	1	0	1	1	0	0	3
25	0	0	0	0	0	1	2	2	2	0	0	7
26	0	0	0	0	1	3	3	6	2	1		16
27	0	0	0	2	7	9	15	3	3			39
28	0	0	0	8	16	28	31	7				90
29	1	3	4	17	25	43	27					120
30	0	4	7	12	36	28						87
Total	1	7	11	39	85	113	78	19	8	1	0	362

*The gray zone of the table corresponds to non-existing values*.

The Apoe4*GM volume interaction term was not statistically significant (ANCOVA: hippocampus: *p* = 0.597; amygdala: *p* = 0.623). A *post hoc* power analysis yield equivalent results for both areas. A total sample of 798 would be needed to achieve 84% power to detect differences among the means vs. the alternative of equal means using an *F*-test with a 0.050 significance level.

### Sub-sample With Amyloid PET

Sixty-four participants underwent an amyloid PET. They did not differ in respect to age, gender, education, MMSE score and continuous cognitive score compared to the 298 cases without amyloid PET (see Table 3).

**Table 3:** Comparison of baseline characteristics of cases with and without amyloid positron emission tomography (PET).

	PET		Total	*P*
	No	Yes		
	*N* = 298	*N* = 64	*N* = 362	
Age at evaluation	74.2 ± 4.1	74.1 ± 4.0	74.2 ± 4.1	0.833
Gender				0.625
F	179 (60.1%)	39 (60.9%)	218 (60.2%)
M	119 (39.9%)	25 (39.1%)	144 (39.8%)
NSC				0.557
<9	50 (16.8%)	11 (17.2%)	61 (16.9%)
9–12	136 (45.6%)	28 (43.8%)	164 (45.3%)
>12	112 (37.6%)	25 (39.1%)	137 (37.8%)
Génotype APOE				0.402
3/2	36 (12.1%)	5 (7.8%)	41 (11.3%)
3/3	212 (71.1%)	47 (73.4%)	259 (71.5%)
4/3	50 (16.8%)	12 (18.8%)	62 (17.1%)
MMSE	28.5 ± 1.3	28.5 ± 1.2	28.5 ± 1.3	0.600
Continuous cognitive	−0.0 ± 3.9	0.2 ± 3.5	0.0 ± 3.8	0.701
score				

Normalized cortical amyloid volume in the hippocampus and the amygdala expressed either as a continuous variable (hippocampus: *p* = 0.714; amygdala: *p* = 0.566) or a binary score (*p* = 0.340; *p* = 0.304) was not associated with cognitive evolution.

## Discussion

Our data in a community-based cohort of highly educated controls indicate reveal that GM densities in the hippocampus and amygdala but not mesial temporal and parietal gyrus are associated with worst global cognitive performances in healthy controls. As recently reported, amyloid load was not related to cognitive changes at follow-up in the present series. This was also the case for white matter hyperintensities (Fazekas score) and ASL values, two MRI variables frequently cited as correlates of cognitive impairment in brain aging (Son et al., 2012; Xekardaki et al., 2015; Arvanitakis et al., 2016; De Vis et al., 2018). In conjunction with previous studies in normal aging, these findings suggest that changes in GM densities in limbic areas is the most reliable correlate of cognitive decline at the very early stages of brain aging.

GM loss in limbic areas has been often associated with time to progression from MCI to AD (Atiya et al., 2003; Kantarci and Jack, 2003; Younes et al., 2014). Early studies have already reported that the decrease of GM density in the hippocampus and amygdala may predict subsequent cognitive decline at the pre-MCI state (Jack et al., 2010; Squarzoni et al., 2012; Edmonds et al., 2015). In the same line, baseline measures of the hippocampus and amygdala in preclinical AD patients predict subsequent development of MCI (Grundman et al., 2002; den Heijer et al., 2006; Griffith et al., 2013; Guderian et al., 2015). Younes and collaborators also reported that hippocampal and amygdala GM atrophy occurs 2–4 years prior to the first signs of AD (Younes et al., 2014). However, the decrease of GM density in these areas varies substantially in preclinical AD cases reflecting the heterogeneity of the structural damage at this stage of the degenerative process (Lauriola et al., 2017; Perrotin et al., 2017). Two recent longitudinal studies in large community-based cohorts led to conflicting data. In the Rotterdam study of 3,264 cases, hippocampal volume was not associated with cognitive decrement at 5 years (Vibha et al., 2018). In contrast, Fletcher et al. (2018) found that baseline hippocampal volumes had significant incremental effects on cognitive decline in 460 cognitively preserved elders. The present data indicate that GM densities in the hippocampus and amygdala are significant predictors of the continuous cognitive score.

Cognitive variations in healthy elders within the normal range may be determined by demographic factors, basal cognition, and APOE genotyping that were not systematically taken into account in multivariate models. In fact, male gender, low level of education, and mainly the presence of APOE4 allele have been associated with worst cognitive trajectories in non-demented elders (Bretsky et al., 2003; Honea R. et al., 2009; Honea R. A. et al., 2009; Zehnder et al., 2009; Haller et al., 2017; Li et al., 2017; Lin et al., 2017) but negative or ambiguous data have also been reported (Van Gerven et al., 2012; López et al., 2017; Min, 2018). In the present study, higher MMSE scores are associated with higher risk of cognitive decline in this series. At first glance, this finding may be surprising. Early contributions showed that single MMSE measures do not allow for identifying MCI subjects who convert to AD (Arevalo-Rodriguez et al., 2015). Moreover, higher MMSE scores at AD diagnosis is associated with faster cognitive decline due to the rapid accumulation of neuropathological changes after diagnosis (Contador et al., 2017). Most of our cases scored 28 and above at baseline. As expected, the vast majority of MMSE 29 and 30 cases showed equal or worst performance at follow-up due to a ceiling effect. Interestingly, the rare cases with MMSE scores of 25 and 26 remained stable or even improved at follow-up pointing to the selection of highly educated cognitively preserved volunteers in this study. From this point of view, our cases cannot be compared to those with NIA-AA preclinical stage 3 that usually display worst cognitive outcomes over time. In respect to APOE genotyping, several studies suggested that the presence of an APOEε4 allele is related to longitudinal changes in medial temporal cortical thickness, and hippocampal atrophy rates (Donix et al., 2010; Lu et al., 2011; Reiter et al., 2017). The independent contribution of APOE genotyping, hippocampal and amygdala atrophy on worst cognitive performances in healthy controls has been suggested by Honea R. et al. (2009) and Squarzoni et al. (2012) in their cross-sectional investigations. However, the association between APOEε4 allele and cognition at the pre-MCI stages is still a matter of debate with some studies showing no association between APOEε4 and memory performances or hippocampal volume in cognitively normal individuals (Protas et al., 2013; Jack et al., 2015; Lupton et al., 2016). When considering the continuous cognitive score that provides an overall assessment of high cortical functions, our data reveal that APOEε4 genotype and GM density in the hippocampus and amygdala are all independent predictors of cognitive decrement in healthy elderly people. Importantly, no such association was found when isolated tests of working or episodic memory were taken into account indicating that detailed neuropsychological exploration of all cognitive abilities in cognitively preserved individuals at baseline where the known patterns of cognitive vulnerability established in MCI could be not applicable.

Our negative data should be also discussed. Adding to recent lines of evidence, PET amyloid load (measured both in neocortical areas as well as hippocampus and amygdala) did not predict subtle cognitive changes in the present cohort (Dubois et al., 2018). Current evidence about the role of amyloid accumulation in cognitively preserved controls remain ambiguous. Although early data suggested that elevated amyloid levels at baseline (SUVr > 1.5) were associated with greater cognitive decline at follow-up (Petersen et al., 2016), more recent contributions indicated that PIB PET β-amyloid’s relationship to cognitive decline was nonlinear being more prominent at lower β-amyloid levels (Knopman et al., 2018). The INSIGHT-pre AD data published recently showed no association between this parameter and cognitive fate at 30-month follow-up in healthy controls (Dubois et al., 2018). Our observations agree with this latter viewpoint implying that the detrimental effect of amyloid accumulation in cognitively preserved elders is at the best marginal. However, this observation should be interpreted with caution since our cases showed low amyloid accumulations with SUVr values >1.2 only in 16/64 of cases. Only two cases exceeded the cut-off value of 1.5. It is thus possible that amyloid burden was too low to produce significant cognitive impact in this cohort. The association of GM densities with cognitive performances is limited to the hippocampus and amygdala and did not involve mesial temporo-parietal association areas. In a previous study, Lancaster et al. (2016) reported that DTI variables in mesial temporal lobe is associated with a decline of episodic memory at 3-year follow-up in healthy controls. In elderly persons with subjective cognitive impairment, Hong et al. (2016) reported decreased fractional anisotropy in supramarginal gyrus and frontotemporal lobes in the absence of GM atrophy. Second, the low to moderate white matter hyperintensities burden (as measured by the Fazekas score) did not impact on the cognitive fate of the present cases. This finding is consistent with a recent study by Moon and collaborators (Moon et al., 2017) who showed that baseline white matter hyperintensities did not predict cognitive decline at follow-up to 3 years in non-demented older adults with memory complaints. Importantly and unlike our cases, the positive association between white matter hyperintensities and neuropsychological decline reported in earlier studies mainly concerned individuals with high Fazekas scores (Son et al., 2012; Arvanitakis et al., 2016). In the same line, ASL values did not predict continuous cognitive score in any of the areas studied. Contrasting with this observation, two recent studies showed that ASL decrease in medial frontal, anterior and posterior cingulate cortex predicts cognitive function in healthy elderly controls (Xekardaki et al., 2015; De Vis et al., 2018). Taken together, these observations imply two distinct MRI correlates of subtle cognitive deficits prior to MCI status: GM loss in the hippocampus and amygdala but also white matter microstructure and brain perfusion changes in neocortical areas outside the mesial temporal and parietal lobes. Such hierarchical pattern of MRI changes in brain aging is consistent with the idea that early GM damage in the hippocampus and amygdala is evident long before the emergence of the very first signs of cognitive failure in brain aging whereas more subtle white matter and functional changes, usually not detected in routine clinical settings, are present in neocortical association areas at the same time period (Younes et al., 2014; Zanchi et al., 2017).

Strengths of the present study include its longitudinal design in a community-based setting, detailed neuropsychological testing at inclusion and follow-up, use of continuous cognitive score that takes into account both improvement and worsening of cognitive performances in each neuropsychological test, consideration of major confounders such as amyloid burden, APOE genotyping and MMSE score at baseline, and inclusion of ASL measures of cerebral perfusion. However, some limitations should also be considered. In the absence of longer follow-up, the decrease of the continuous cognitive score does not represent a marker of incipient dementia. No CSF measures of tau and Aβ protein were available in this work so that the real extent of AD pathology remains unknown. Our multivariate model explains only 7% of the cognitive variability. When interpreting this modest percentage, one should keep in mind that, in contrast to MCI and AD cases, healthy controls display an impressive variability in MRI parameters (Lauriola et al., 2017; Perrotin et al., 2017). The combination of multiple MRI modalities including ASL and DTI data in other neocortical areas but also CSF or PET assessment of tau pathology is warranted to improve the performance of imaging-based models of cognitive prediction in normal brain aging.

## Data Availability

The datasets generated for this study are available on request to the corresponding author.

## Ethics Statement

The protocol was approved by the Ethics Committee of the Geneva University Hospitals of Geneva. All experimental procedures were carried out in accordance with the approved guidelines and with the principles of the Declaration of Helsinki. All participants were given written informed consent prior to inclusion.

## Author Contributions

FH, VG, SH and PG: conceived the study. CR, M-LM and SH: recruited. CR, M-LM and PG: neuropsychology supervising. VG, M-LM and SH: imaging. CR, M-LM and FH: data preparation. FH, CR, SH, VG, M-LM and PG: analyzed the data and manuscript writing.

## Conflict of Interest Statement

The authors declare that the research was conducted in the absence of any commercial or financial relationships that could be construed as a potential conflict of interest.

## Footnotes

^1^http://www.fmrib.ox.ac.uk/fsl/

^2^http://fsl.fmrib.ox.ac.uk/fsl/fslwiki/BET

^3^http://fsl.fmrib.ox.ac.uk/fsl/fslwiki/fast

^4^www.ncss.com
